# Evaluation of Self-vision Assessment Charts in Schools of Eastern Nepal: A Multi Method Study

**DOI:** 10.31729/jnma.8796

**Published:** 2024-11-30

**Authors:** Archana Shrestha, Sudhir Kumar Thakur, Abhishek Roshan, Sanjay Kumar Singh, Archana Pokhrel, Apekshya Bohara, Lisasha Poudel, Chanda Thakur, Asmita Adhikari, Priyanka Timsina, Yunika Acharya, Rajiv Ranjan Karn

**Affiliations:** 1 Department of Public Health, Kathmandu University School of Medical Sciences, Dhulikhel, Kavre, Nepal; 2 Lahan Eye and Ear Care System, Sagarmatha Choudhary Eye Hospital, Lahan, Siraha, Nepal; 3 Institute for Implementation Science and Health, Kathmandu, Budhanilkantha, Nepal; 4 Department of Community Programs, Dhulikhel Hospital Dhulikhel, Kavre, Nepal

**Keywords:** *awareness*, *ocular*, *school*, *vision*

## Abstract

**Introduction:**

Globally, over two billion people suffer from vision impairment, almost half preventable. In Nepal, the Eastern Regional Eye Care Program introduced self-vision assessment charts in 137 schools for early eye issue detection. This study assessed the charts' use and perceived impact.

**Methods:**

A multi-method study was conducted to evaluate the use of self-vision assessment charts in schools in Eastern Nepal. Ethical approval was obtained from Institutional Review Board (Reference number: 72-079-040). Quantitative data were collected through a questionnaire survey. Quantitative data were analyzed using descriptive statistics, while qualitative data were analyzed using framework analysis. Both methods were integrated to enhance understanding of participants' experiences.

**Results:**

In this study, 400 students participated in questionnaire survey, 180 in self vision assessment, and 16 teachers in in-depth interviews. Among the participants, 198 (49.50%) of students used the chart, 125 (65.78%) expressed satisfaction, 241 (60.25%) found it useful, 276 (69%) as beneficial, and 253 (63.25%) stated improved health. There were 93 (23.25%) students who felt knowledgeable about its use, with 18 (10%) using it correctly. In 162 (90%) of schools there were adequate chart placement, 23 (12.77%) provided clear signage for viewing points. Teachers recognized the chart's benefits and encouraged its use, but students lacked awareness. Regular use was viewed as beneficial for early detection of eye issues, though challenges included a need for more awareness among students and teachers and reliance on home remedies.

**Conclusions:**

Most students and teachers acknowledged self-vision assessment charts' benefits but had low utilization and accuracy rates. Strategic interventions such as regular informational sessions, teacher motivation, and engagement with eye health experts are essential.

## INTRODUCTION

Globally, more than two billion people suffer from vision impairment, with about half of these cases being preventable.^1,2^ In Nepal, a study reported that uncorrected myopia affects 15.2% of urban and 8.2% of rural students.^3^ School-based interventions, like selfvision assessment, offer a cost-effective solution for the early detection and prevention of eye problems.^4,5^ However, awareness about eye health among children remains low, resulting in untreated issues that can affect their education and future opportunities. To address blindness prevention, the Eastern Regional Eye Care Program (EREC-P) introduced self-vision assessment charts–simple, accessible tools for individuals to check their vision–which Sagarmatha Choudhary Eye Hospital painted on the walls of 137 schools in eastern Nepal, along with easy-to-understand instructions. These charts guide students and teachers in monitoring their vision and seeking eye care if necessary. This study evaluated the use and impact of these charts in the Siraha, Sarlahi, and Udayapur districts and gathered teachers' perceptions of their effectiveness in improving future eye health initiatives in schools.

## METHODS

A multi-method study was conducted to evaluate the utilization and adoption of self-vision assessment charts displayed on school walls in Eastern Nepal. The quantitative component included a questionnaire survey among students and observations of students using the vision charts. The qualitative component involved in-depth interviews with teachers. The data was collected from March to September 2022. The Ethical approval was obtained from the Institutional Review Board (Reference number: 72-079-040).

For the quantitative survey, the study participants were students. Inclusion criteria for students were those attending the selected schools in grades 6-12 and were present at the time of data collection. The sample size calculation for the quantitative component of the study was calculated by following formula:


$$\begin{array}{ll} \text{n} & ={\text{Z}}^{2}\times \frac{\text{p}\times \text{q}}{{\text{e}}^{\text{2}}}\\ & =1.96^{2}\times \frac{\text{0.50}\times \text{0.50}}{0.05^{2}}\\ & =384 \end{array}$$

Where, Z = 1.96, p = 50% = 0.5, q = 50% = 0.5 Assuming p of study 50%

q = 1-0.5 = 0.5 (q=1-p), allowable error (e) = 5%

Using these parameters, the estimated sample size for the study was calculated to be 384; it was rounded to a total of 400.

A multistage sampling approach was employed to select districts, schools, and students. Initially, we randomly selected three out of the six districts (Sarlahi, Udayapur, Siraha) where the program was implemented. Then, two schools from each district were randomly selected. Finally, a pretested, selfadministered questionnaire was distributed to 66 students from each school. The questionnaire was developed through a structured process, incorporating relevant literature and expert input to ensure content validity. It was designed to capture comprehensive information on socio-demographic characteristics, utilization, and perceptions of the self-vision assessment chart. Pretesting of the questionnaire was conducted in a similar population to refine the questions for clarity and comprehensiveness. Feedback from this pretest was utilized to revise the instrument, enhancing its readability and relevance to the target population.

The quantitative questionnaire was designed to collect comprehensive data on (a) socio-demographic characteristics, including age, gender, ethnicity (classification as per Demography Health Survey)^6^, and religion; (b)the utilization of the self vision assessment chart, focusing on the frequency of use, satisfaction with the chart, and whether friends utilized it; and (c) students' perceptions of the chart, including its effectiveness, accessibility, and their willingness to share knowledge about it with peers . Responses were rated on a five-point Likert scale, ranging from "Very dissatisfied" to "Very satisfied." Additionally, observations were conducted to asses the placement and visibility of the self-vision assessment chart, considering factors such as the clarity and understandability of the letters, the presence of signs for measuring distance, and the appropriate positioning of the chart being assessed. Students' ability to use the chart correctly was also assessed by observing adherence to specific procedures: maintaining the correct distance while checking vision, closing one eye to read the letters, attempting to read up to the last letter, and following all instructions accurately among a total of 180 randomly selected students (30 from each school).

The study participants for the qualitative component included school teachers who met specific inclusion criteria. Adult teachers aged 18 years or older who were actively working in the schools were included, while those on long-term leave were excluded to ensure consistent and relevant insights. Teachers who could provide valuable perspectives on the effectiveness of the self-vision assessment chart were purposively selected. Qualitative data were gathered through in-depth interviews, using a semistructured, open-ended questionnaire that allowed for flexibility in responses and an in-depth exploration of the teachers' perceptions. The qualitative interview guide focused on gathering insights into participants' perceptions and experiences regarding the selfvision assessment chart, including knowledge of the chart, including information sources and personal experiences; persuasion examing perceptions of the chart's relative advantage and compatibility; benefits, health contributions, and appropriateness; the complexity of using the chart was investigated by understanding students' feelings about its ease of use; trialability through initial experiences; observability concerning the chart's placement, and the decision factors influencing usage, including motivations and challenges; implementation patterns and the confirmation of the chart's long-term impact on vision health, seeking supporting reasons and examples. This structured approach facilitated a comprehensive understanding of participants' experiences with the chart. All interviews were conducted in the Nepali language and in a private space. Each interview was audio-recorded and transcribed verbatim.

Quantitative data were analyzed using STATA-14. Participants' characteristics were presented using frequencies and proportions for categorical variables, while means and standard deviations (SD), median, Interquartile Range (IQR) were reported for normally distributed numerical variables. For qualitative data, framework analysis was conducted.^7^ A codebook was developed based on the study's objectives and the diffusion of innovation theory^8^, which includes stages such as Knowledge, Persuasion, Decision, Implementation, and Confirmation. Deductive analysis was employed, utilizing pre-defined codes derived from the theoretical framework to guide the coding process.

## RESULTS

The study included 400 participants of which 201 (50.25%) were male and mean age of participant was 16.09±1.70 years (Table 1).

Among the 16 teachers, who participated in qualitative interviews, male were 11 (68.75%), the mean age was 37.4 ± 10.26 years, with all teachers having at least a bachelor's degree. The mean duration of work experience 7.63± 5.13 years (Median:10, IQR: 5-15) and 13 (81.25%) were secondary-level teachers.

There were 198(49.5%) of the students who had previously used the chart. The mean frequency of utilization was 1.86 ± 1.5 times (Median: 1, IQR: 0.51.5). Among those who used the chart, 125 (65.72%) were satisfied (including those who were very satisfied), (Table 2).

A total of 241 students (60.25%) agreed (including those who totally agreed) that the chart is useful for checking their eyes, and 276 (69%) felt that the chart could be beneficial, while 253 (63.25%) agreed that it improves their health. Similarly, 268 (67%) of students also consented to the chart's compatibility with their eye-checking preferences. In terms of accessibility, 238 (59.5%) reported that they can access the chart anytime, and 242 (60.5%) found easy to use. There were 82 (20.5%), who found the chart difficult to use. Ninty three (23.25%) felt they have enough knowledge to use it, and 175 (43.75%) could it effectively, indicating a knowledge gap. Peer support is positive, with 201 (50.25%) helping each other, and 213 (53.25%) observing benefits for others. (Table 3).

**Table 1:** Characteristics of students participating in self-administered questionnaire survey (n= 400).

Characteristics	Male (n=201)	Female (n=199)	Total (%) (n=400)
**Ethnicity**			
Brahmin/Chhetri	47 (23.36)	70 (35.17)	117 (29.25)
Dalit	16 (7.95)	5(2.51)	21 (5.25)
Janjati/Aadiwasi	49 (24.38)	42 (21.11)	91 (22.75)
Madhesi	83 (41.29)	80 (40.20)	163 (40.75)
Others	6 (2.98)	2 (1.01)	8 (2.00)
**Religion**			
Hindu	172 (85.57)	181 (90.95)	353 (88.25)
Buddhist	17 (8.46)	13 (6.53)	30 (7.50)
Muslim	6 (2.98)	1 (0.50)	7 (1.75)
Christian	1 (0.50)	1 (0.50)	2 (0.50)
Others	5 (2.48)	3 (1.51)	8 (2.00)
**Grade**			
Grade 6	6 (1.49)	2 (1.01)	8 (2.00)
Grade 8	26 (12.93)	23 (11.56)	49 (12.25)
Grade 9	84 (41.79)	69 (34.68)	153 (38.25)
Grade 10	54 (26.87)	55 (27.64)	109 (27.25)
Grade 11	29 (14.43)	41 (20.60)	70 (17.50)
Grade 12	6 (2.98)	5(2.51)	11 (2.75)

**Table 2:** Utilization of self-vision assessment Chart amongst students (n= 400).

Utilization	n (%)
Use of self-vision assessment chart before	198 (49.5)
Frequency of using self-vision assessment chart to test the vision (Mean±SD)	(1.86±1.5)
Level of satisfaction among those who used (n = 190)	
Not satisfied	18 (9.47)
A little satisfied	8 (4.21)
Neutral	39 (20.52)
Satisfied	92 (48.42)
Very satisfied	33 (17.36)

**Table 3:** Perception of students on self-vision assessment chart (n=400).

Perception of self-vision assessment chart	Totally disagree n(%)	Disagree n(%)	Neutral n(%)	Agree n(%)	Totally agree n(%)
Checking my eyes through a self-vision assessment chart will be good.	22 (5.5)	10 (2.5)	127 (31.75)	139 (34.75)	102 (25.5)
I think self-vision assessment charts are beneficial.	15 (3.75)	7 (1.75)	102 (25.5)	170 (42.5)	106 (26.5)
Use of self-vision assessment contributes to my better health	16 (4)	12 (3)	119 (29.75)	160 (40)	93 (23.25)
Use of a self-vision assessment chart is compatible with my eye checking preferences.	15 (3.75)	32 (8)	85 (21.25)	180 (45)	88 (22)
I possess the knowledge required to use self-vision assessment chart	50 (12.5)	140 (35)	117 (29.25)	67 (16.75)	26 (6.5)
I can use a self-vision assessment chart effectively with my existing knowledge	29 (7.25)	79 (19.75)	117 (29.25)	127 (31.75)	48 (12)
I can access a self-vision assessment chart any time I like.	20 (5)	50 (12.5)	92 (23)	169 (42.25)	69 (17.25)
I first try self-vision assessment chart and then use it.	24 (6)	10 (2.5)	-	190 (47.5)	115 (28.75)
It is difficult for me to use a self-vision assessment chart.	68 (17)	152 (38)	98 (24.5)	58 (14.5)	24 (6)
It is easy for me to use a self-vision assessment chart.	28 (7)	43 (10.75)	144 (36)	130 (32.5)	55 (13.75)
It is easier for me to access a self-vision assessment chart whenever I need to check my eyes.	19 (4.75)	51 (12.75)	88 (22)	165 (41.25)	77 (19.25)
					
I can observe that the use of a self-vision assessment chart for eye checking purposes benefits those around me.	9 (2.25)	41 (10.25)	137 (34.25)	141 (35.25)	72 (18)
I can tell others regarding the benefits of self­vision assessment charts.	32 (8)	72 (18)	115 (28.75)	129 (32.25)	52 (13)
I can share about the process to use the self-vision assessment chart to my friends	28 (7)	92 (23)	122 (30.5)	112 (28)	46 (11.5)
My friends help each other out to use self-vision assessment chart	28 (7)	67 (16.75)	104 (26)	120 (30)	81 (20.25)

**Table 4:** Observation of accuracy in using the chart and its placement by students (n=180).

Observation	n (%)
**Accuracy in utilizing self vision chart**	
Maintained correct distance (6 meters) from the self vision assessment chart	26 (14.44)
One eye covered to read out letters	115 (63.89)
Tried to read up to the last letter	108 (60)
Same procedure followed for another eye	72 (40)
Tried to read up to the last letter	65 (36.11)
Participants who test his/her eye in the correct way	18 (10)
**Placement of the charts**	
Good spot for placement of the chart.	162 (90)
The letters written on the wall were clear and understandable.	136 (75.56)
The sign was clear and understandable.	23 (12.78)

The observation results indicate that 26 (14.44%) maintained the correct distance of six meters from the self-vision assessment chart. There were 115 (63.89%) participants, covered one eye while reading the letters. However, only 72 (40%) repeated the procedure for the other eye. Ultimately, only 18 (10%) used the chart correctly. In terms of chart placement, 162 (90%) charts were in good locations, 136 (75.56%) letters were clear and understandable, but only 23 (12.78%) schools had clear signage for viewing points (Table 4).

Teacher's perception on the self assessment vision chart:

**Knowledge:** All interviewed teachers were familiar with the self-vision assessment Chart and could accurately explain its proper use. Several respondents (n=8) highlighted the chart's usefulness for students in self-testing their vision and improving their eyesight. They noted that it aids in detecting vision defects such as myopia and hyperopia, which previously required visits to Lahan. With the charts, students can gain insights into their vision without needing to go to the hospital needing to go to the hospital because of the charts. Additionally, three respondents mentioned that they provide students with information on how to use the chart and actively encourage them to utilize it.

*“Through this self vision, students can determine the condition of their eyes, and find out if they have shortsightedness or long-sightedness. Earlier, we had to reach Lahan for this but it's not the case anymore"* ***-Siraha_07, Sec.teacher, 26years, M***

**Awareness:** Four respondents noted a lack of awareness among students regarding the importance of the self vision assessment chart and its proper usage. Two students had not received information about the chart following its placement. One respondent pointed out that some students overlooked the chart, either due to ignorance or because they did not perceive any eye problems. Additionally, another respondent mentioned that only older students had been informed about the chart, leaving younger students unaware of its existence.

*"Many students do not know or use the chart now.. even though they were excited initially... they don’t care about it... due to our ignorance or might be due to lack of information"* ***-Udayapur_04, Sec.teacher, 50years, F***

*"Students didn't know the purpose of the self-vision chart or how to use it properly. Some simply ignored it, thinking they had no eye issues.* ***-Udayapur_04, Sec.teacher, 50years, F***

**Perceived usefulness:** All respondents agreed that the chart is effective and beneficial for students. They highlighted its utility in enabling students to assess their own eye conditions, identify potential vision issues, and prevent eye diseases. Additionally, they noted that the chart has raised awareness about dietary habits essential for eye health and the significance of proper eye care. Three respondents indicated that they encourage students to use the chart if they have any concerns, subsequently involving parents and referring children to eye care facilities as needed. One respondent underscored the chart's importance for timely access to health services and treatment.

According to the respondents, students frequently use the chart during leisure periods or tiffin time, and parents have also taken an interest in it. Furthermore, teachers actively encourage students to utilize the chart. Five respondents recognized the eye as a crucial sense organ essential for health, emphasizing the importance of eye care for overall well-being and acknowledging that initiatives like the chart raise students' awareness on how to improve eyesight and address eye problems, ultimately benefiting their health.

*“If doubtful, we make students assess their eyes using this chart; if it's still doubtful, we call the guardians and refer the students"* ***- Siraha_03, Principal, 45 years, M***

*“If we take care of our eyes well, it will have a positive impact on our health; it will strengthen our health"* ***- Sarlahi_04, Sec.teacher, 45years, M***

**Compatibility:** Nine respondents indicated that the process of using the self-vision assessment chart is user-friendly, free of charge, and easily understood by everyone. Several respondents mentioned that the chart's process mirrors that of assessments conducted in hospitals. However, one respondent expressed that two individuals should be involved in the assessment–one to evaluate vision and another to provide support. Most agreed that there are no negative aspects to the assessment process, though four suggested the need for regular follow-ups, reporting, and frequent eye check-up camps for students. One respondent described how students who identified vision defects through the chart sought consultation with doctors at eye camps or Katari Eye Hospital and received appropriate treatment, including necessary medications and glasses.

*“It's a good system.I practically used it and made students try it too; it works properly and detects eye defects of students"* ***-Udayapur_01, Sec.teacher, 35 years, F***

*“It's a good process.they can observe the picture and analyze their vision by themselves.it's a good thing"* ***- Sarlahi_01, Sec.teacher, 23years, F***

**Complexity:** Nine respondents indicated that the process of using the self-vision assessment chart is user-friendly. One participant noted positive feedback from students, who recognized the chart's benefits and engaged in discussions about its use with their parents and teachers. However, two respondents highlighted the importance of counseling and encouragement for students, emphasizing that awareness of the chart's benefits is crucial for long-term usage.

*"it’s a good process, easy way, simpie...anybody can understand”* ***-Siraha_03, Principal, 45years, M***

*“Process is easy, I asked students about it. they gave good feedback.everyone has been finding it to be good, we have been making them use the chart at times"* ***- Udayapur_01, Sec.teacher, 35 years, F***

**Utilization:** Five respondents expressed that students exhibited excitement and curiosity when they encountered the self-vision assessment chart for the first time. Those who successfully read all the letters felt happy and relieved, while those who struggled recognized the need to seek help at Lahan Hospital. One respondent emphasized the chart's utility for all students, particularly benefiting those from economically disadvantaged backgrounds. They reported that students were initially excited and used the chart effectively, but its usage has declined. One respondent noted that he had not observed any students using it independently.

*"initially the students used it a iot taking turns.they seemed happy.it was difficult to get turns, now they just don’t care about it"* ***-Udayapur_04, Sec.teacher, 50 years, F***

**Observability:** Regarding the placement and positioning of the chart, most respondents indicated that its location is suitable, accessible, and easily visible to all. Three respondents noted that the placement was determined through careful discussion and research. Some respondents emphasized that one chart suffices for the school, explaining that students take turns using it, with different classes testing their eyesight at designated times.

*"it’s in a good place because students can see it any time.it’s visible while praying, everyone sees it, we can easily understand it...it’s good"* ***-Siraha_01, Sec.teacher, 30 years, M***

*"it’s suitable, i guess.it’s an invisible place, easy to use for aii"* ***-Sarlahi_02, Sec.principal, 50 years, M***

Two respondents indicated that the chart's position should be lowered slightly for younger students. One respondent expressed concern about the chart and viewing point not being uniformly leveled within the school. Several suggested that placing the chart directly in front of the entrance would allow parents to see it as well. Additionally, some respondents recommended having multiple charts to enhance convenience for student use.

*"The positioning is good for students of class5 and above but it might be a little bit higher for lower class students"* ***-Udayapur_04, Sec.teacher, 50 years, F***

**Perceived benefits:** Nine respondents acknowledged the benefits of using the chart systematically and regularly. They noted that consistent utilization could enhance students' knowledge, prioritize health, facilitate the timely detection of eye problems, and promote the prevention and treatment of eye diseases. Three respondents committed to maintaining its use, highlighting that students have become increasingly health-conscious and are likely to continue utilizing the chart. One respondent expressed noticing students using the chart regularly and that it has been advantageous for the school.

*"Through regular utilization of this chart, they can understand the situation of their eyes, they will know about their eye problems if there’s any.and addressing it by going for a checkup, using glasses so, i think it’s effective"* ***-Sarlahi_02, Principal, 50 years, M***

**Challenges:** Nine respondents believed that there were no obstacle or disadvantages associated with using the chart. However, four highlighted challenges, including a lack of awareness and students regarding the chart's purpose and usage, two respondents had insufficient demarcation of the viewing point, and one respondent lack of regular follow-ups from relevant authorities. They also noted potential issues, such as students becoming overly conscious about their eye health, excessive use of the chart, and resorting to home remedies instead of seeking professional eye care. Additionally, one respondent emphasized the importance of guiding younger students in using the chart, as they may not fully understand its purpose.

*"We did not encourage students to use the self vision assessment chart because we were not aware about its usefuiness"* ***-Sarlahi_01, Sec.teacher, 23years, F***

**Perception on scale-up:** Two respondents (suggested that the nationwide implementation of this program could lead to several benefits, including heightened awareness of eye care, prevention of eye diseases, and timely recognition and treatment of eye issues, particularly in rural areas. Two respondents noted that, given the program's advantages and the growing concern surrounding eye health, it will likely gain acceptance and support from all stakeholders.

*"Eye related problems are increasing day by day... students from different places attend schools.if this kind of program of self assessment is expanded in aii the schools throughout the country, everyone will be able to check their eyes and know how safe they are...*

*it would be even better to implement it everywhere* ***- Udayapur_02, Sec.teacher, 40 years, M***

**Suggestions:** Respondents proposed several strategies to enhance the effectiveness of the intervention, including conducting frequent informative sessions for teachers and students about the chart, providing demonstrations, ensuring regular follow-ups, reporting and feedback mechanisms, maintaining the chart, clearly marking the viewing points, organizing regular eye check-up camps for students, adding more charts, and integrating relevant information into the curriculum. Additionally, some suggested (n=3) organizing regular awareness programs at the community level, while another respondent highlighted the potential benefits of involving specialists in the program for follow-up.

*"Not just for schools, this should be implemented at the community level for those children deprived of education...if there are any eye defects they can get the information.not just for schools, it should be implemented everywhere where there's human settlement"* ***-Siraha_07, Sec.teacher, 26 years, M***

## DISCUSSION

The study assessed the self-vision assessment charts installed in schools in six districts in eastern Nepal. About 49.5% had used it previously, and 65.72% reported satisfaction with the chart. While 59.9% of students acknowledged its usefulness for eye checks and 69% saw potential benefits, only 23.3% felt knowledgeable about using it, and about 59.6% reported easy access. Only about 10% used the chart correctly, following all procedures correctly. While 90% of schools had adequate chart placement, but only 12.8% featured clear signage for viewing points. Most teachers are familiar with the self-vision assessment Chart and recognize its utility in enabling students to self-test their vision and identify issues such as myopia and hyperopia without needing to visit hospitals. Despite this, students lack awareness about the chart's importance and proper usage, leading to decreased interest over time. Many respondents noted the chart's effectiveness in promoting eye health awareness and facilitating timely treatment. While the chart is generally seen as user-friendly, there is a need for increased counseling and support to ensure ongoing usage. Observations indicate that the chart is well-placed in schools, although some adjustments for younger students could enhance accessibility. Most respondents acknowledged the benefits of regular use for health education, yet some challenges persist, including insufficient awareness and potential overreliance on home remedies rather than professional care. Suggestions for improvement include frequent informational sessions, regular follow-ups, and broader community outreach to raise awareness about eye health and the chart's benefits. The respondents believe expanding the program nationwide could significantly enhance eye care awareness and disease prevention, particularly in rural areas. Studies have shown that, such self-vision testing programs have been effective in identifying common vision problems early, raising awareness, and improving access to care, especially in underserved populations.^3,9-11^ The World Health Organization (WHO) has also advocated for school-based vision screening programs as a means to identify vision problems early and ensure timely referrals to healthcare providers.^12^

The results reveal a mixed level of engagement and awareness regarding the self vision assessment charts among students. Approximately 49.5% of students reported having previously used the chart, indicating that nearly half of the target population is somewhat engaged, yet there remains room for increased utilization. While utilization was suboptimal, it suggests that with proper training, secondary school students could effectively use the vision self-assessing tool. Similar findings were shown from a Nigeria study; about 40.5% of the students used the vision corridor in four weeks.^13^ A similar study conducted in India among school children aged 10 to 19 years reported a utilization rate of 99%.^9^ This higher rate may be attributed to the established school eye health program in that region.

Only 23.3% of the students confirmed having knowledge about it. This data partially aligns with qualitative findings, as some teachers also expressed uncertainty about the chart and acknowledged a lack of information among students. In this study, only about 10% of students were able to accurately follow the procedure for measuring their vision. While we did not compare their accuracy against expert assessments, findings from a Nigerian study indicated a perfect level of agreement (Kappa = 1) between self-assessed visual acuity and assessments made by trained ophthalmic assistants after a week of orientation to the student.^13^ This suggests that, despite the low accuracy in following measurement procedures among students in our study, the self-assessment process may still align closely with the assessments of trained professionals if awareness about accurate measurement is increased. Another study from Nepal showed the effective screening of visual acuity by trained students with a high sensitivity of 82% and specificity of 98%.^14^

In most schools, the letters displayed on the wall were clear and easily understandable. Additionally, most schools had effectively positioned the chart in an accessible and visible locations. However, the signs were only apparent and understandable in a few schools. These findings align with qualitative data indicating that most teachers recognized the importance of appropriate chart placement and positioning. Conversely, several teachers reported inadequate demarcation of viewing points, which may stem from a lack of follow-up by the relevant authorities, resulting in insufficient chart maintenance. Furthermore, shortcomings in the orientation and demonstration processes may have contributed to students' lack of knowledge regarding the standard procedures for using the chart.

Qualitative interviews highlighted the chart's perceived benefits as an awareness-raising tool facilitating timely access to eye health care. Most respondents found the Self Vision Assessment Chart user-friendly, free of charge, and easily understandable, with several noting that its process resembles hospital assessments. Teachers identified their and parents' roles in motivating students to increase the use of the chart and see better eye care. In this context, a study in China found a positive association between teacher-parent communication and students' pursuit of vision care.^15^ Similarly, a study in Canada identified advice from friends and family as a significant motivator for seeking eye care.^16^

Most respondents felt there were no obstacles or disadvantages to using the chart; however, some highlighted challenges, such as a lack of awareness among teachers and students about its purpose and usage, inadequate signs of the viewing point, and insufficient follow-ups from relevant authorities. Concerns were raised about students potentially becoming overly conscious about their eye health, excessively using the chart, relying on home remedies instead of seeking professional care, and the need for guidance for younger students. Respondents proposed several strategies to enhance the intervention's effectiveness, such as conducting frequent informative sessions and demonstrations for teachers and students, ensuring regular follow-ups, and clearly marking viewing points on the chart. Additional suggestions included organizing regular eye checkup camps, incorporating relevant information into the curriculum, holding community awareness programs, and involving specialists for follow-up support.

Our study has several strengths. To our knowledge, this is the first study in Nepal that utilized a multimethod design to evaluate the utilization and adoption of self vision assessment charts in Eastern Nepal, combining quantitative surveys of students with qualitative interviews of teachers. This allowed us to gather comprehensive data on socio-demographic characteristics, chart usage, and perceptions of its effectiveness among students and in-depth insights into teacher's perspectives, facilitating a deeper understanding. However, this study acknowledge some limitations. First, the quantitative component is based on self-reported data from students. Second, the observational assessments of chart placement and usage were conducted at a single time, which may not reflect ongoing conditions or changes in the school's environment. Finally, the qualitative interviews were limited to purposively selected teachers and may not have reflected the views of all teachers in these schools.

## CONCLUSIONS

This study demonstrates a positive response to the implementation of visual assessment charts in schools, with many students reporting satisfaction and health benefits from their use. However, proper utilization remained limited, as only a small proportion of students knew how to use the charts correctly. While most schools had adequate placement of the charts, clear signage for viewing points was often insufficient. Teachers acknowledged the charts' benefits and promoted their use, but a lack of awareness among students and reliance on home remedies hindered their full potential. To address these challenges, the study suggests implementing strategies like educational sessions, community awareness programs, and involving specialists for follow-up care to improve awareness and effectiveness.
